# Correlation of hair and plasma efavirenz concentrations in HIV-positive South Africans

**DOI:** 10.4102/sajhivmed.v20i1.881

**Published:** 2019-04-29

**Authors:** Jenna Johnston, Lubbe Wiesner, Peter Smith, Gary Maartens, Catherine Orrell

**Affiliations:** 1 Division of Clinical Pharmacology, Department of Medicine, University of Cape Town, Cape Town, South Africa; 2 Desmond Tutu HIV Centre, Institute of Infectious Disease and Molecular Medicine, Cape Town, South Africa; 3 Department of Medicine, University of Cape Town, Cape Town, South Africa

**Keywords:** Adherence, Antiretroviral therapy, Hair, Plasma, Drug concentrations

## Abstract

**Background:**

Antiretroviral concentrations in hair provide a longer window of drug detection and are useful for measuring longer-term drug exposure. Efavirenz is an important component of first-line treatment in resource-limited settings, but its concentrations in hair have not been well studied.

**Methods:**

This study is a supplementary to a randomised controlled trial of an adherence intervention using an electronic adherence measuring device. Hair and plasma samples were collected from human immunodeficiency virus-positive patients in Cape Town, South Africa. Previously validated liquid chromatography tandem mass spectrometry methods were used to measure efavirenz concentrations in the collected hair and plasma samples. *CYP2B6* genotyping of participants was also performed. Data analysis was performed using descriptive and comparative statistics as well as regression modelling.

**Results:**

Hair samples were collected from 59% of patients enrolled in the parent study. Results indicated that hair efavirenz concentrations were significantly influenced by participants’ *CYP2B6* metaboliser status. Median efavirenz concentrations for extensive, intermediate and slow metaboliser genotypes were 3.54 ng/mg, 5.11 ng/mg and 10.66 ng/mg, respectively. A strong correlation was observed between the efavirenz concentrations measured in hair and plasma samples (Spearman’s correlation coefficients, 0.672–0.741, *p* < 0.0001). No relationship between hair efavirenz concentrations and virological failure or adherence measured using an electronic adherence was shown.

**Conclusion:**

The results from this study provide further insight into the potential of using hair as a matrix for measuring antiretroviral concentrations. However, challenges experienced in collecting hair samples suggest that this adherence measure may have limited utility in an African population.

## Introduction

For antiretroviral therapy (ART) to be successful in preventing disease progression, high levels of adherence are required.^1,2^ Methods for measuring adherence include patient self-report, pill counts, pharmacy refill records, electronic drug monitoring and therapeutic drug monitoring (TDM). TDM involves the determination of drug or drug metabolite concentrations in plasma and, more recently, hair.^3^ Each of these methods has both advantages and disadvantages which have been discussed in detail in the literature.^1,2,4,5,6^ There remains no gold standard for determining adherence.^7^

TDM is a direct method of monitoring adherence by measuring drug exposure. Drug concentrations in plasma provide a short-term (up to a few days) assessment of drug exposure, whereas drug concentrations in hair provide a longer window of drug detection (weeks to months).^3^ There are benefits in analysing both plasma and hair samples because, while a low plasma concentration indicates recent poor drug exposure, a low hair concentration indicates average poor drug exposure for the previous month, as suggested by Van Zyl et al.^8^ Additionally, a high plasma concentration in combination with a low hair concentration could provide insight into the adherence patterns of patients and identify cases where medication is being taken just prior to clinic visits. Little data have been published comparing the relationship between antiretroviral (ARV) drug concentrations in these two matrices.^8,9^

Currently, the most preferred ART for first-line treatment in resource-limited settings includes efavirenz (EFV) in combination with tenofovir and either emtricitabine or lamivudine.^10^ There is only one other study that has reported data comparing EFV concentrations in hair and plasma, in which concentrations of EFV in hair were shown to be strongly correlated with 24-h intensive pharmacokinetic measurements and only weakly correlated with single plasma measurements.^11^ Plasma EFV concentrations are characterised by high inter-individual variability in concentrations, which can be explained, in part, by polymorphisms in the *CYP2B6* gene that has been reported to influence EFV metabolism.^12^ High plasma EFV concentrations have been associated with central nervous system side effects,^13^ which can lead to patients discontinuing treatment. Hair concentrations of EFV are also influenced by *CYP2B6* genotype.^14,15^

This study is a supplementary to our recently reported randomised controlled trial of an adherence intervention^16^ and investigates the potential of measuring EFV concentrations in hair to monitor adherence. In addition to determining the effect of *CYP2B6* metaboliser status on hair EFV concentrations, the relationship between plasma and hair EFV concentrations will be assessed. Lastly, the relationship between the adherence measured by an electronic adherence monitoring device (EAMD) and hair EFV concentrations will be explored.

## Methods

### Setting and participants

The parent study was a randomised controlled trial over 48 weeks in ART-naïve individuals, which showed that SMS reminders triggered by real-time EAMD had little impact on cumulative adherence to ART.^16^ Participants were recruited from a large outpatient ART centre in Gugulethu, Cape Town – the Hannan Crusaid Treatment Centre (HCTC). ART-naïve adults and adolescents (≥ 15 years old) were eligible for the parent study if they were commencing treatment at the HCTC, had their own mobile phone and were willing to sign an informed consent form. The details of the parent study have been described elsewhere.^16^

### Sub-study design and participants

Participants recruited for the parent study were given the option of participating in the sub-study if the hair on their head was longer than 1 cm. The participants involved provided samples of hair at weeks 16, 32 and 48.

### Measures and analyses – Laboratory procedures

The measures collected for the parent study^16^ and the previously reported pharmacokinetic and pharmacogenetic sub-study^17^ are described subsequently. In the parent study, adherence was monitored using a Wisepill^®^ device,^18^ a real-time EAMD. In a related study, the EAMD was shown to be the best adherence measure to predict virologic outcomes.^19^ The EAMD is of the size of a mobile phone and can store up to a week of medication in a seven-compartment pill box. Every participant received an EAMD, and when each time the device was opened, a signal was sent via the mobile phone network to a secure central computer, thereby recording tablet taking or treatment interruptions in real time. Any recorded opening on a day during the study was classified as an adherent day, and cumulative adherence was calculated as the number of adherent days divided by the number of days in care.

Blood was drawn for HIV-1 viral load (HIV-1 RNA 3.0 assay^®^; Bayer Healthcare, Leverkusen, Germany) at screening and at weeks 16 and 48. Additional blood was drawn for mid-dosing interval EFV concentrations (in the time window between 9 h and 16 h after self-reported EFV intake) at weeks 16, 32 and 48. Three *CYP2B6* loss-of-function single-nucleotide polymorphisms associated with EFV concentrations were chosen and analysed: rs3745274 (516G→T); rs28399499 (983T→C); and rs4803419 (15582C→T). Based on their *CYP2B6* genotype, participants were classified as either slow, intermediate or extensive metabolisers using a simplified version of Holzinger’s^20^ metaboliser status classification.

For the determination of EFV concentrations, blood samples were centrifuged at 3500 rpm for 10 min. Plasma was transferred into labelled cryovials that were frozen at -80 °C until analysis. Plasma EFV concentrations were determined by a liquid chromatography/tandem mass spectrometry (LC-MS/MS) method validated for the concentration range 0.0195–20 µg/mL. Hair samples, collected at weeks 16, 32 and 48, were analysed at the same laboratory for EFV using a validated LC-MS/MS method.^21^ The method was validated for the concentration range 0.625–40 ng/mg. The centimetre of hair closest to the scalp was analysed to represent drug exposure of approximately one month.

### Statistical analysis

Descriptive data were summarised using median and interquartile range (IQR) for continuous data and percentages for categorical data. The Χ^2^ test was used to compare proportions, and the two-tailed *t*-test (for normal variables) or the Mann–Whitney test (for skewed variables) was used to compare continuous variables. GraphPad Prism 4 (California, USA) was used for the statistical analysis of data, including Spearman’s correlation. Regression modelling was used to explore whether EFV hair concentrations were associated with the adherence measured using the EAMD and virological outcome. Sex, baseline CD4 cell count, metaboliser status and age were included as independent variables. Samples that were determined to be below the assay methods limit of quantification were analysed as 0.624 ng/mg for EFV concentrations in hair and 0.0194 µg/ml for EFV concentrations in plasma.

## Ethical consideration

Ethical approval for the study was given by the University of Cape Town, Faculty of Health Sciences, Human Research Ethics Committee (HREC/REF: 101/2015). Informed consent was provided by each of the study participants. The parent clinical trial was registered in the Pan African Clinical Trials Registry (number PACTR201311000641402).

## Results

### Baseline characteristics

Of the 230 individuals enrolled in the parent study, 135 individuals provided hair samples. The majority, 92.6%, of this subset cohort were Black African females. A total of 257 hair samples were collected from the 135 individuals, consisting of 93 at week 16, 75 at week 32 and 89 at week 48. On average, two hair samples were collected from each of the individuals who participated in the sub-study. A comparison of the baseline demographics for the cohort and the subset is detailed in Table 1.

**TABLE 1:** Baseline demographics of the subset compared to the total cohort.

Variable	Cohort						Subset						*p*
	*n*	%	mean	s.d.	Median	IQR	*n*	%	mean	s.d.	Median	IQR	
Number	230	-	-	-	-	-	135	-	-	-	-	-	-
Female sex	150	65.2	-	-	-	-	125	92.6	-	-	-	-	< 0.0001
Age (years)	-	-	34.5	9.1	-	-	-	-	33.7	8.9	-	-	0.338
Weight (kg)	69	-	69	15.1	-	-	-	-	71.6	15.1	-	-	0.312
Height (cm)	-	-	164	8.6	-	-	-	-	161.2	7	-	-	0.019
CD4 count (cells/mm^3^)	-	-	-	-	225.5	131.5–287	-	-	-	-	234.5	152.5–290.5	0.266
Log HIV RNA (copies/mL)	-	-	-	-	4.9	4.4–5.4	-	-	-	-	4.7	4.4–5.3	0.114

s.d., standard deviation; IQR, interquartile range; HIV, human immunodeficiency virus; RNA, ribonucleic acid.

### Cohort and subset adherence

High levels of adherence were observed in the subset. The median adherence of the subset was higher at weeks 16 and 48 compared to that of the cohort. Of the 230 individuals enrolled in the parent study, only 160 individuals returned for the week 16 visit, for blood sampling, and only 180 at week 48. These individuals provide the adherence comparison for the subset with hair samples (Table 2).

**TABLE 2:** Cumulative electronic adherence monitoring device adherence at weeks 16 and 48 for the cohort and subset.

Variable	Week 16			Week 48		
	Adherence %			Adherence %		
	*n*	Median	IQR	*n*	Median	IQR
Cohort^19^	160	93	74–98	180	86	59–94
Subset	93	100	92–100	89	101	96–107

EAMD, electronic adherence monitoring device; IQR, interquartile range.

Few individuals enrolled in the subset displayed virologic failure: at week 16, 4 out of 93 (4.3%) individuals had viral loads greater than 400 copies/mL, and at week 48, 5 out of 89 individuals (5.6%) had viral loads greater than 50 copies/mL.

### Hair efavirenz concentrations according to *CYP2B6* metaboliser status

**FIGURE 1:**
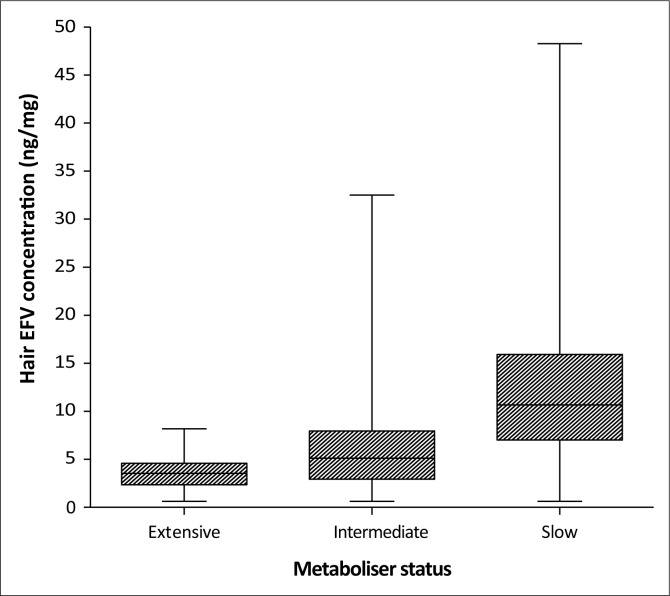
Median (interquartile range) efavirenz (EFV) concentrations in hair according to *CYP2B6* metaboliser status. Hair EFV concentrations determined at weeks 16, 32 and 48 were combined for the above analysis.

Results from the genotyping of the participants indicated that out of the 135 participants who provided hair samples, 34 (25.2%), 61 (45.2%) and 35 (25.9%) were extensive, intermediate and slow metabolisers, respectively, similar to the parent trial cohort.^17^ The metaboliser status for five of the participants was missing. The EFV concentrations measured in the hair samples were analysed according to the metaboliser status for each of the participants and are presented in Figure 1. There was a significant difference between the median EFV concentrations in hair for each metaboliser status (ANOVA, 95% CI, *p* < 0.0001). Median EFV concentrations for the extensive, intermediate and slow metaboliser genotypes were 3.54 ng/mg (IQR: 2.35–4.59), 5.11 ng/mg (IQR: 2.93–7.94) and 10.66 ng/mg (IQR: 7.01–15.93), respectively.

### Relationship between efavirenz concentrations in hair and plasma

**FIGURE 2:**
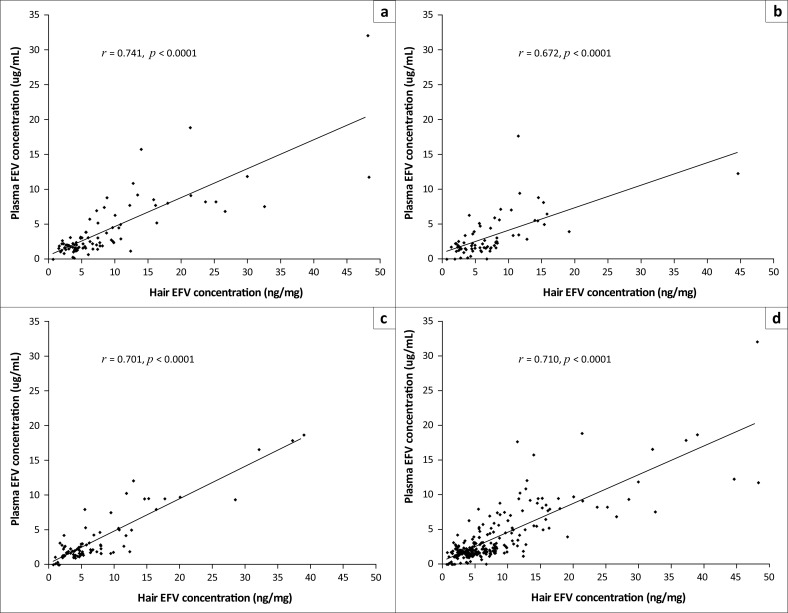
Scatterplots showing the correlation between plasma and hair efavirenz (EFV) concentrations at weeks 16 (a), 32 (b), 48 (c) and for all weeks combined (d). Spearman’s correlation coefficients used to assess the relationship between EFV concentrations in hair and plasma are shown.

Scatterplots of the correlation between hair and plasma EFV concentrations are presented in Figure 2. Spearman’s correlation coefficients were used to assess the relationship between the concentrations of EFV in the two matrices. The results indicate that hair and plasma EFV concentrations were strongly correlated at all three sampling time points in the study (correlation coefficients, 0.672–0.741; all *p*-values < 0.0001).

### Hair efavirenz concentrations as a predictor of adherence and virological outcomes

**FIGURE 3:**
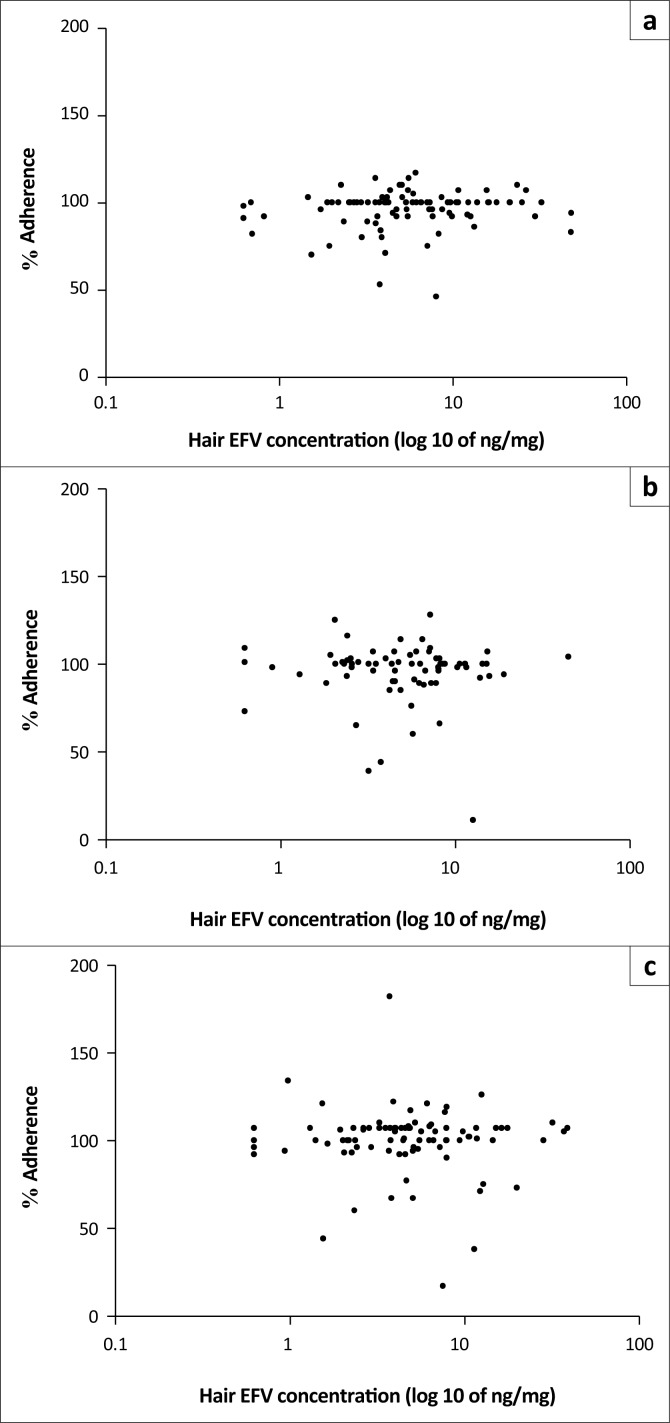
Scatterplots showing the relationship of adherence assessed by electronic adherence monitoring device (EAMD) (represented as a percentage) to concentrations of efavirenz (EFV) in hair at weeks 16 (a), 32 (b) and 48 (c).

The relationship between adherence measured by the EAMD and hair EFV concentrations is presented in Figure 3. Regression analysis showed that the only variable with significant impact on EFV hair concentration in this cohort was metaboliser status (data not shown). Age, sex and adherence did not significantly alter EFV hair concentration.

Very few individuals in the study cohort experienced virological failure; and, in a similar regression model, no variables, including hair EFV concentrations, had an impact on virological failure (data not shown).

## Discussion

In this adherent study cohort, hair and plasma EFV concentrations were highly correlated. Hair samples were provided by participants who were both retained in care and willing to donate a head hair sample. Data from the parent study showed that a high percentage of the participants in this cohort who provided pharmacokinetic samples were virally suppressed.^17^ We were unable to show an association between hair EFV concentrations and virological failure, likely because of the low occurrence of virological failure in the study cohort. There was no relationship between hair EFV concentrations and adherence measured by EAMD, which we have previously shown was the best adherence measure (compared with pharmacy refills, pill counts, self-report and plasma EFV concentrations) for predicting virological failure and resistance development.^19^ However, lack of correlation between adherence measures does not imply that the measures do not predict outcomes as there is no gold standard adherence measure.

Hair EFV concentrations are significantly influenced by participants’ *CYP2B6* metaboliser status. A similar result was reported when the corresponding plasma samples, collected for the related sub-study, were analysed according to metaboliser status.^17^ Participants with the slow metaboliser genotype displayed significantly higher median EFV concentrations in both short- and long-term EFV exposure. This observation is consistent with the results reported in an earlier study where individuals with the slow metaboliser genotype were shown to have greater than threefold increases in EFV concentrations in both plasma and hair samples.^15^ Besides this study, one other study has also investigated the influence of *CYP2B6* polymorphisms on hair EFV concentrations.^14^ Hair samples were collected from HIV-infected women in South Africa, and results from the study also showed increased concentrations of EFV in hair samples from individuals with the slow metaboliser genotype.^14^

Plasma and hair EFV concentrations were strongly correlated throughout the 48-week study period (Figure 2), which suggests that, for our cohort with very high virologic suppression, a single plasma concentration was as good an adherence measure as a single hair concentration. This is an interesting result as hair concentrations provide an average level of drug exposure over the last 30 days or so,^22^ whereas single plasma concentrations represent a brief snapshot of drug exposure and are also subject to ‘white coat effects’ (good adherence around the time of clinic visit).^23^ As a result, it is suggested in the literature that hair ARV concentrations might be of more value than single plasma concentrations when measuring adherence.^3,22,24^ Because virologic failure was uncommon in our study, we were unable to evaluate either adherence measure as a predictor of virologic outcomes.

Hair EFV concentrations were found to have a non-significant association with adherence measured by the EAMD (Figure 3). These results suggest that, for this study, no significant correlation between adherence and EFV concentrations in hair exists. Even though the median adherence measured by the EAMD was 100% at weeks 16 and 32 (IQR: 92–100 and 92–103, respectively) and 101% (IQR: 96–107) at week 48, the concentration of EFV in the hair samples varied, most likely because of the different metaboliser genotypes present in the subset. While hair ARV concentrations have previously been shown to correlate well with virological suppression,^8,24,25,26,27,28^ this has not been the case when the relationship between hair concentrations and measures of adherence such as self-report and EAMD have been assessed.^11,29,30^ As previously discussed, the low occurrence of virological failures in this subset did not allow for hair EFV concentrations to be associated with virologic outcomes for this study. The relationship between hair EFV concentrations and virologic outcomes needs to be further explored in a cohort with a higher rate of virological failures.

The study was limited by the study design, in that the participants who provided hair samples were predominantly female, had higher levels of adherence compared to the rest of the cohort and were mostly virologically suppressed. This limited the extent to which the data could be analysed and did not allow for certain associations to be investigated. The collection of head hair samples was also challenging as many women were reluctant to donate a braid and most men had shaved heads. Only 135 (59%) of the 230 participants that were enrolled in the parent study provided hair samples.

## Conclusion

In conclusion, we have shown that a strong correlation exists between EFV concentrations measured in plasma and hair samples collected from participants with good ARV adherence. In addition, patient metaboliser status was observed to have a significant effect on long-term exposure to EFV. However, because of the low rates of virologic failure in our cohort, we were unable to assess the ability of EFV concentrations in hair to predict outcomes. The challenges we experienced in collecting hair from our cohort suggest that this adherence measure may have limited utility in an African population.
